# A High Sensitivity IDC-Electronic Tongue Using Dielectric/Sensing Membranes with Solvatochromic Dyes

**DOI:** 10.3390/s16050668

**Published:** 2016-05-10

**Authors:** Md. Rajibur Rahaman Khan, Alireza Khalilian, Shin-Won Kang

**Affiliations:** School of Electronics Engineering, Kyungpook National University, 80 Daehakro, Bukgu, Daegu 41566, Korea; rajibur@ee.knu.ac.kr (M.R.R.K.); alireza.khalilian991@gmail.com (A.K.)

**Keywords:** electronic tongue, interdigitated capacitor, solvatochromic dye, sensing membrane, sensing element, dielectric constant, response time

## Abstract

In this paper, an electronic tongue/taste sensor array containing different interdigitated capacitor (IDC) sensing elements to detect different types of tastes, such as sweetness (glucose), saltiness (NaCl), sourness (HCl), bitterness (quinine-HCl), and umami (monosodium glutamate) is proposed. We present for the first time an IDC electronic tongue using sensing membranes containing solvatochromic dyes. The proposed highly sensitive (30.64 mV/decade sensitivity) IDC electronic tongue has fast response and recovery times of about 6 s and 5 s, respectively, with extremely stable responses, and is capable of linear sensing performance (*R*^2^ ≈ 0.985 correlation coefficient) over the wide dynamic range of 1 µM to 1 M. The designed IDC electronic tongue offers excellent reproducibility, with a relative standard deviation (RSD) of about 0.029. The proposed device was found to have better sensing performance than potentiometric-, cascoded compatible lateral bipolar transistor (C-CLBT)-, Electronic Tongue (SA402)-, and fiber-optic-based taste sensing systems in what concerns dynamic range width, response time, sensitivity, and linearity. Finally, we applied principal component analysis (PCA) to distinguish between various kinds of taste in mixed taste compounds.

## 1. Introduction

An electronic tongue/taste sensor array is a smart/intelligent instrument with an appropriate pattern recognition system, which can detect different types of taste from simple or complex mixtures of soluble nonvolatile molecules in a sample [1]. Taste sensors have huge applications in research institutes, pharmaceutical industries, food and beverage industries, medical diagnostic centers, environmental monitoring centers, and the agricultural sector [2,3,4,5,6,7,8,9].

Several different measuring/detecting techniques have been proposed in the literature for developing electronic tongues, such as voltammetry [10,11,12,13,14,15,16,17,18], amperometry [19,20], and potentiometry [21,22,23,24].

A disposable multichannel screen-printed lipid-based taste sensor was proposed by Sim *et al.*, to control the quality of milk [25]. The advantages of this taste sensor are its small size, simple construction, and low cost, however, it has a response time of approximately 60 s. Thete *et al.* developed an optochemical fluorometric microspot array to detect various kinds of alcoholic beverages [26]. The main features of this sensor array include being disposable and having low fabrication cost, but its sensing mechanism is complex, and the sensor is bulky. Jeong Hyun-Min *et al.* developed a Cascoded Compatible Lateral Bipolar Transistor (C-CLBT) taste sensor with a lipid/polymer membrane as a sensing membrane; the sensor was operated in a MOSFET/BJT hybrid-mode. This sensor has the advantage of presenting a linear response, but it also has several disadvantages such as the need for an external electrode (making the sensor bulky), and a low sensitivity with very low dynamic range (1 fM to 1 mM), which stops it from matching the human taste threshold level [27].

An electronic tongue to detect various tastes using a surface acoustic wave [28] was proposed by Sehra *et al.* The operation and construction of this electronic tongue is simple, but the main disadvantage is its lack of selectivity. A fiber-optic multichannel taste sensor based on the evanescent wave absorption principle was developed by Lee *et al.* [29]. The developed sensor can detect five basic tastes with high sensitivity, is easy to fabricate, and it has a response time of approximately 10 s; however, it also has various demerits, such as short dynamic range, poor reproducibility and reliability issues. Sohna *et al.* proposed an electronic tongue using polymer beads in a microfluidic channel, to make a capillary-based tongue [30]. Their proposed electronic tongue has several advantages: low cost, small size, and real-time response. It also has various shortcomings, such as a complicated fabrication procedure, the need for a light source, and a charge-coupled device.

Much research has been directed to the use of interdigitated electrode (IDE) structures in a vast number of applications, such as biosensors [31,32,33,34,35,36,37], dielectric studies on thin films [38], gas sensors [39,40,41], pH sensors [42], bacteria detection [43], medical applications [44], optically-controlled microwave devices [45], humidity [46,47,48,49,50,51], pressure sensors [52], tunable devices [53], and chemical sensors [54].

An impedance spectroscopy taste sensor using gold interdigitated electrodes (IDEs) was developed by Riul *et al.* [55], with a composition of different chemical materials in an ultrathin membrane form deposited onto an IDE. The merits of this developed taste sensor are that the sensing units do not need any electroactive materials, and that it does not need a reference electrode. The impedance measuring mechanism of the sensor is, however, based on a complicated sensing instrument, with the impedance being observed by changing the frequency of a signal employed in the sensor’s electrode terminal.

We propose a highly sensitive interdigitated capacitor (IDC) electronic tongue with wide dynamic range, fast response and recovery time, low-cost, and high stability, to detect several kinds of taste, for example sweetness (glucose), saltiness (NaCl), sourness (HCl), bitterness (quinine-HCl), and umami (monosodium glutamate). The proposed electronic tongue has four IDC sensing elements. Four different kinds of solvatochromic dye (Nile red, Auramine O, Reichardt’s dye (R-dye), and Rhodamine B) were separately mixed with polyvinyl chloride (PVC) polymer and a N,N-dimethylacetamide (DMAC) solution, to prepare four different kinds of dielectric/sensing solutions. These four dielectric solutions were separately deposited into IDEs using a spin coater, to make the four different types of IDC taste sensing elements of the array. We believe this is the first time that an IDC taste sensor array/electronic tongue using a solvatochromic-dye based sensing membrane is reported. The proposed solvatochromic-dye based IDC taste sensor array/electronic tongue operates on a capacitance variation principle. When the IDC sensing elements of the electronic tongue is dipped into the taste-solution, the sensing membrane dielectric constant changes because of the charge transfer character of the solvatochromic dye molecules in it; this change in the sensing membrane’s dielectric constant modifies the IDC capacitance, which will then originate a variation in the received sensing signal’s amplitude. A data acquisition (DAQ) module was used to collect information from each channel of the IDC sensor array. We developed a LabVIEW program to collect the taste information from the array, and save the sensing information in an associated computer. The proposed IDC electronic tongue has other advantages, including real time monitoring capability, high reproducibility, and linear sensing performance. We applied an easy and very efficient data processing method, principal component analysis (PCA), to discriminate various tastes from a mixed set of taste samples, and obtained excellent performance. 

## 2. Theory and Principle of Operation of the Electronic Tongue System

### 2.1. Electronic Tongue System Analogy

**Figure 1:**
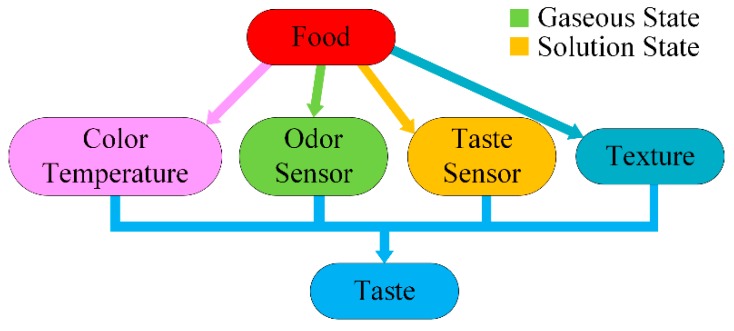
Quantification of food palatability.

Sensory evaluation of palatable foods is more than a mere question of taste. It is also influenced by different kinds of other human senses such as sight, smell, touch, and hearing; this is illustrated in Figure 1. The notion of taste includes five basic qualities: sweetness, saltiness, sourness, bitterness, and umami. The Japanese term umami is used for “palatability”, a savory sensation caused by the monosodium glutamate (MSG) contained in disodium guanylate in mushrooms, seaweeds, and disodium inosinate in fish and meat [56,57,58]. Different chemical compounds characterize the different types of basic taste, and are tabulated in Table 1.

**Figure 2:**
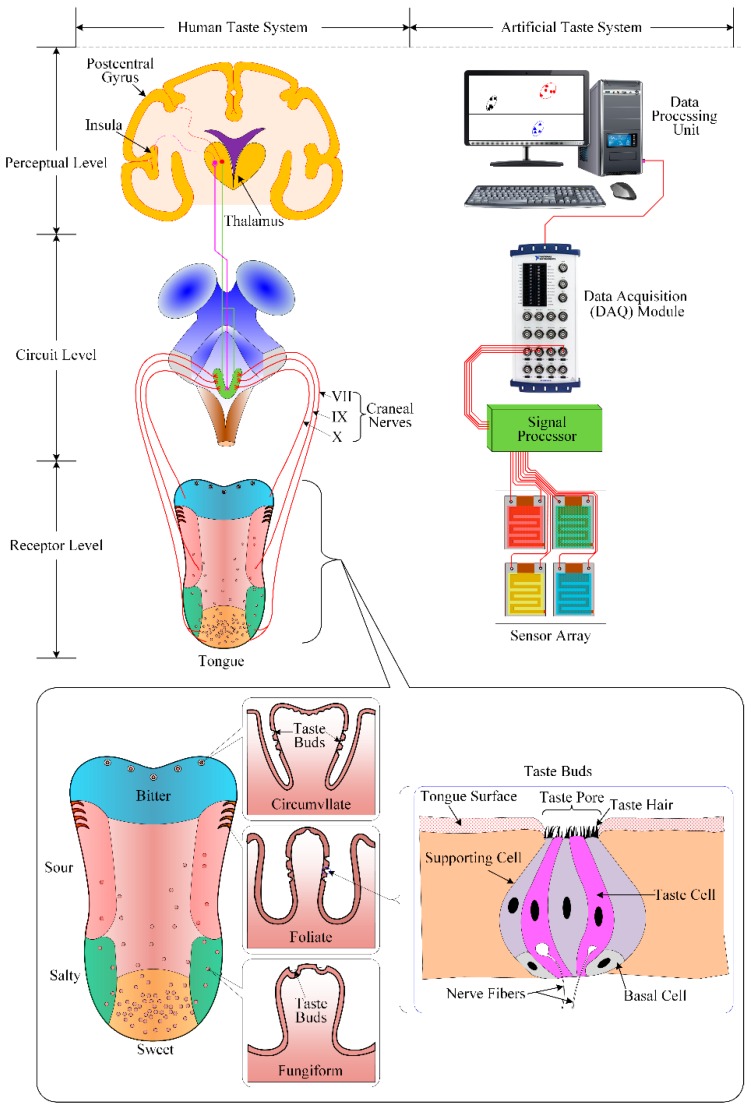
Functional analogy within the human (biological) taste sensing system and the electronic taste sensing system.

When addressing the issue of food taste detection, analogies can be set up within the biological taste sensing system as well as the electronic taste sensing system, insofar as several approximations can be establish in their operating principles and structure; these analogies are presented in Table 2 and Figure 2.

The surface of the human tongue has many small projections (known as papillae), inside which the taste buds are located. Taste buds are flask-like in shape, consisting of 50–100 individual taste cells joined together in a spherical structure. At the opposite end, taste cells are connected with a network of nerve fibers.

The human/biological tongue offers different sensitivities at different regions: the tip of the tongue is most sensitive to sweetness and saltiness, the sensation of sourness is best sensed on the lateral sides of the tongue, and the maximal sensitivity to bitterness appears on the back of the tongue. To feel the taste of food, the taste pores in the oral cavity absorb the chemicals of food. The taste cells in the taste buds collect these chemical stimuli and create nerve impulses. The taste impulses from the tongue are first transferred to specialized cells in the brain stem by two nerves (the facial nerve and the glossopharyngeal nerve) for initial processing. The taste impulses then ascend to the thalamus. After further analysis in the thalamus [59,60], the taste information is relayed to the part of the cerebral cortex that intervenes in the conscious appreciation of taste. The sensorial information about temperature and texture from the tongue triggers also a cortex response [61].

In the case of an electronic tongue, the sensor array consists of different sensing elements. Each sensing element has maximal sensing abilities for a particular taste, even though it can also respond slightly to other different tastes. When the taste molecules/chemicals react with the taste sensitive membranes of the sensing elements, the membranes’ electrical properties change, altering the received sensing signal. The electrical signals from various sensing elements of the array are transferred into the signal processing unit. After being processed in the data acquisition/signal processing unit, the converted signals are transferred into the data processing unit. This unit is an important part of the electronic tongue system; each one of the several sensing elements of the array generates a complicated response in a multi-component environment. Therefore, a relevant multidimensional data processing unit is needed; various pattern recognition methods have been applied for this purpose, such as PCA, Artificial Neural Networks (ANN), Partial Least Squares (PLS), and several others [62,63,64,65,66,67,68,69,70]. These pattern recognition methods aim to recognize the sensed taste, categorize between different kinds of tastes, and classify tastes into given sets.

### 2.2. Theoretical and Mathematical Formulation of the Proposed IDC Electronic Tongue

**Figure 3:**
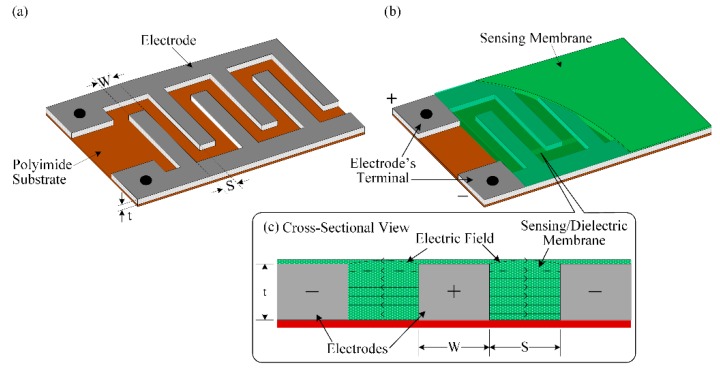
Schematic diagram of the interdigitated electrode: (**a**) without the dielectric/sensing membrane; (**b**) dielectric/sensing membrane in the IDE; and (**c**) cross-sectional view of the IDC.

An interdigitated capacitor (IDC) is constituted by a special type of electrodes designed in a finger/comb shape with a periodic interlocking pattern and deposited on a substrate; these electrodes are then covered with a dielectric film, as shown in Figure 3. We applied an AC voltage v at two terminals of IDC to produce an electric field within the electrodes. The electric field flux lines then penetrate the dielectric, from the positive electrode to the negative one. The unit cell capacitance per length of an interdigitated electrode with an isotropic dielectric film material is given by [71]:

(1) $$C_{\text{UC}}=\varepsilon_{0}\varepsilon_{r}K\left(\frac{\sqrt{1-{\left(\text{S}/\lambda \right)}^{2}}}{K\left(\text{S}/\lambda \right)}\right)+2\varepsilon_{0}\varepsilon_{r}\left(\frac{t}{S}\right)$$

where $\varepsilon_{0}$ is the absolute and $\varepsilon_{r}$ is the relative dielectric constants of the sensing membrane. S is the space between two adjacent fingers of the IDE, t is the electrode thickness, and λ is the spatial wavelength.

The spatial wavelength λ is defined as [72,73]:

(2) λ=2(W+S)

where W is the width of the electrode.

The total IDC capacitance can be calculated by:

(3) $$C=L\left(\text{N}-1\right)\left\{\frac{\varepsilon_{0}\varepsilon_{r}}{2}\frac{K\left(\sqrt{1-{\text{k}}^{2}}\right)}{\text{K}(\text{k})}+2\varepsilon_{0}\varepsilon_{r}\left(\frac{t}{S}\right)\right\}$$

where N is the number of fingers of the IDE, and L is their length. The parameter K(k) represents the elliptic integral of the first kind of modulus k. The modulus k is defined as:

(4) $$k=\cos\left(\frac{\pi W}{\lambda }\right)$$

Therefore, from Equations (2) and (4) we get:

(5) $$k=\cos\left(\frac{\pi }{2}.\frac{W}{\text{S}+\text{W}}\right)$$

where:

(6) $$\text{K}(\text{k})=\int_{0}^{1}\frac{\text{K}(\text{k})}{\left(\sqrt{\left(1-{\text{t}}^{2}\right)\left(1-{\text{k}}^{2}t^{2}\right)}\right)}\text{dt}$$

The peak value of the voltage across the IDC sensing element ($v_{C}$) can be written as follows:

(7) $$v_{C}=\frac{i_{C}}{2\pi fC}$$

where $i_{C}$ is the peak value of the current flowing through the IDC sensing element of the array and f is the signal frequency. The change in the IDC sensing element’s voltage due to the change in the taste solution’s concentration (and the resulting vary in the IDC capacitance) can thus be written as:

(8) $$\Delta v_{C}=\frac{i_{C}}{2\pi f\Delta C}$$

## 3. Experimental Work

### 3.1. Fabrication Procedure of the Interdigitated Electrode

**Figure 4:**
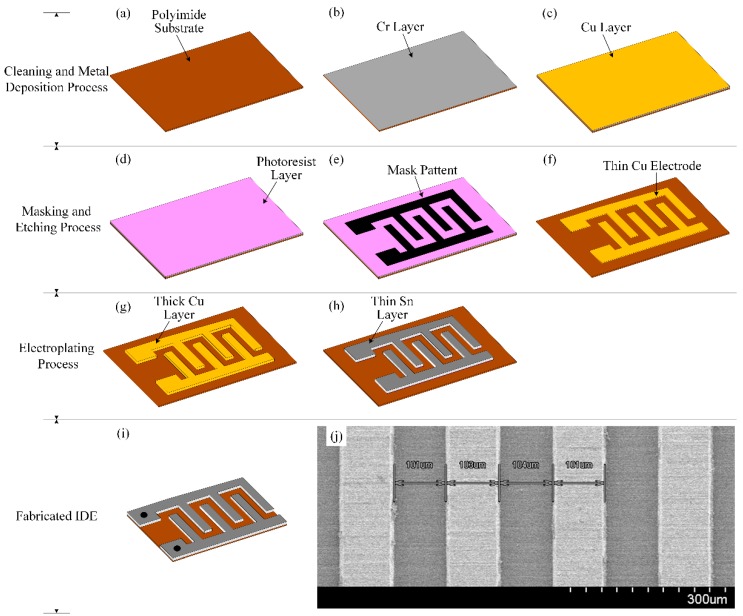
Preparation procedure of the IDE: (**a**) polyimide substrate; (**b**) thin Cr layer on the polyimide substrate; (**c**) depositing a thin Cu layer on the Cr layer; (**d**) photoresist layer, (**e**) transferring the mask pattern onto the photoresist layer; (**f**) etching the unmasked metal layers; (**g**) depositing the thick Cu layer onto the thin patterned Cu layer via electroplating; (**h**) depositing the thin Sn layer onto the thick patterned Cu layer; (**i**) cutting the residual polyimide substrate; and (**j**) SEM image of the surface of the interdigitated electrode (IDE).

We prepared the interdigitated electrode (IDE) with 40 pairs of fingers by vacuum evaporation and an electroplating deposition process on a 4 × 2 cm polyimide (PI) substrate with a thickness of approximately 22 µm. First, we deposited thin Cr and Cu layers (approximately 10 nm and 15 nm thick, respectively) on the PI substrate by vacuum evaporation. Then we transferred the photo mask of the IDE pattern onto the deposited metal layer and etched the unmasked patterned with a chemical etchant, to obtain the thin IDE fingers on the PI substrate. We then used a Cu electroplating process to increase the thickness of the Cu electrodes. Finally, a thin Sn layer was deposited on the thick Cu electrodes, and the residual PI substrate was cut. The step-by-step preparation procedure of the IDE is illustrated in detail in Figure 4. A scanning electron microscope (SEM) (S-4800, Hitachi, Ibaraki, Japan) was used to determine the IDE thickness, the finger’s width, and the widths of the gaps between fingers; values of approximately 22 μm, 100 μm and 100 μm were obtained, respectively.

### 3.2. Fabrication Procedure of Interdigitated Capacitor (IDC) Sensing Elements

The capability of a chemical compound to change color due to variations in the polarity of a solvent is defined as solvatochromism. Solvatochromism can be positive or negative [74,75]; a dye with solvatochromic properties is called a solvatochromic dye. In our experiments, we used a sensing/dielectric membrane containing a solvatochromic dye, to form the array of IDC sensing elements. In our experiment, we used four different IDC sensing elements in the array. To make these four different sensing elements, we selected four different types of solvatochromic dyes (Nile red [76,77], R-dye [78], Auramine O [79], and Rhodamine B [80,81]), a polymer (PVC), and DMAC as solvent. We bought all chemicals from the Sigma-Aldrich Chemical Corporation (Seoul, South Korea) and used them without any purification.

**Figure 5:**
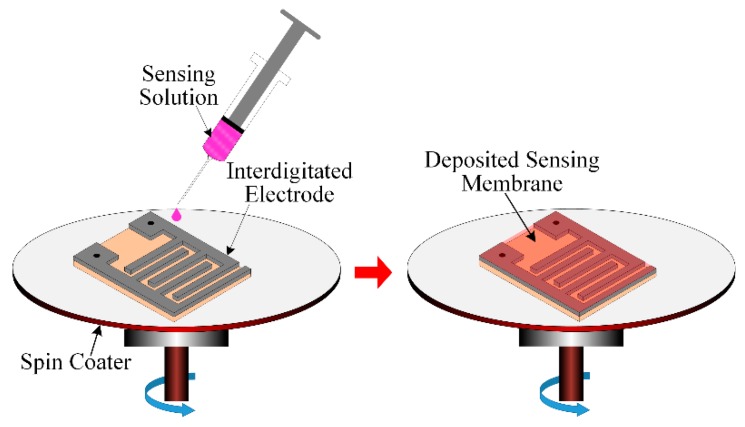
Deposition process of the sensing/dielectric solution into the IDE with a spin coater, to obtain the IDC.

Four different types of sensing/dielectric solution in denser liquid form were prepared, and placed individually onto the IDE, thus making the IDC sensing elements of the array. Table 3 shows the chemical composition of four different sensing/dielectric solutions for the IDC sensing elements (S1 to S4) of the electronic tongue. The four types of sensing solutions were prepared with the following process: First, 0.014 g of every solvatochromic-dye was separately mixed into 8 mL of DMAC and sonicated for 10 min to prepare a dye-solution. Then, 0.056 g of PVC was mixed into dye-solution and sonicated for 10 min, to get the final sensing/dielectric solution for the IDC. To properly wash the IDE we used acetone, methanol, and deionized (DI) water, respectively; the IDE was then dried with N_2_ gas. After that, we deposited the sensing solution into the IDE with a spin coater, and dried the IDC at room temperature. This step-by-step fabrication process is shown in Figure 5.

### 3.3. Detection Mechanism of the Proposed IDC Electronic Tongue

**Figure 6:**
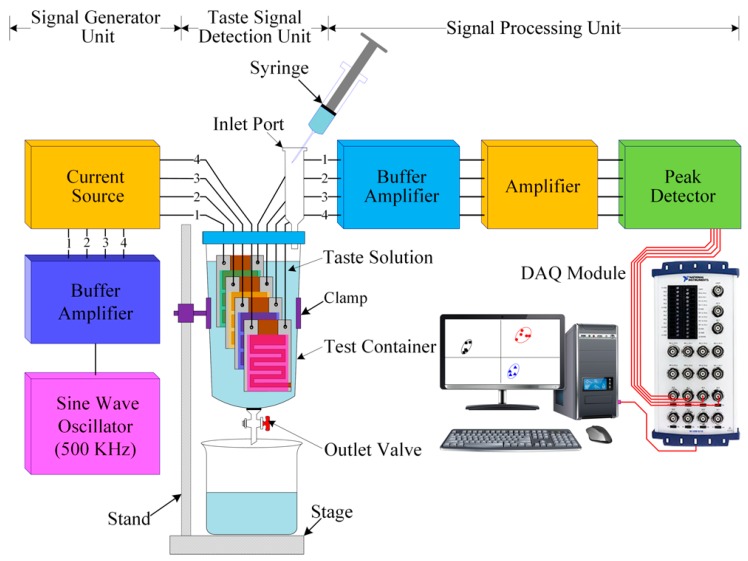
Schematic diagram of the proposed electronic tongue’s experimental setup.

Figure 6 shows a schematic diagram of the designed IDC electronic tongue’s experimental setup. This setup involves three units: a signal generator unit, a taste signal detection unit, and a signal processing unit. The signal generator unit is a 500 kHz sine wave oscillator, whose output is linked to the input of a four channel buffer amplifier (to avoid loading effects). The four outputs of the buffer amplifier are linked to the inputs of four controllable current generators. The taste detection unit consists of four IDC sensing elements and a test chamber made of Teflon, to prevent reactions between the target taste solution and the test chamber. The four inputs of the IDC sensing elements are connected to the outputs of the four current sources. 

When a voltage is applied across the IDC sensing element of the array, an electric field is generated that penetrates the dielectric/sensing material. If the taste solution’s concentration changes, then the dielectric constant of the IDC sensing elements will change accordingly, and as a result the output voltage of each IDC sensing element of the array will also change. The outputs of the IDC sensor array are connected to the inputs of the signal processing unit, which consists of a buffer amplifier, a voltage amplifier, and a peak detector [82,83,84,85]. The signals received from the sensor array are first fed to the high input impedance buffer amplifier, and afterwards to the voltage amplifier, for adequate voltage amplification. The peak detectors are used to convert the amplified received ac sensing signals to dc voltages; their outputs are connected to the DAQ module inputs. In our experiment, we used five different kinds of taste: sweetness (glucose), saltiness (NaCl), sourness (HCl), bitterness (quinine-HCl), and umami (monosodium glutamate). Different types of taste substances were individually mixed with DI water to get the desired concentrations of the five taste solutions (1 µM to 1 M). The IDC sensor array was vertically installed into the test container. During measurement, we closed the outlet valve and slowly injected approximately 15 mL of the target (taste or reference) solution into the test chamber with a syringe, using the inlet port. After measuring the concentration of the taste solution, we opened the out port to remove the taste solution from the test chamber and clean the container. 

The principle of operation of the designed sensor array is based on capacitance variations. As discussed, when the IDC taste sensor array contacts the taste solution, the sensing membrane’s dielectric constant of the IDC sensing element change, thus changing the IDC capacitance. As a result, the voltage across the IDC and, hence, the output voltage of the peak detector change as well. At first, we injected DI water into the test container to get a reference voltage; the target taste solution was then injected, to obtain the corresponding sensing voltage; the difference between those two voltages is defined as the relative voltage (ΔV) of that particular taste solution. The DAQ module was interfaced with the signal processing unit and the computer to collect sensing information from the array and store the collected data in the computer for further processing.

## 4. Results and Discussion

**Figure 7:**
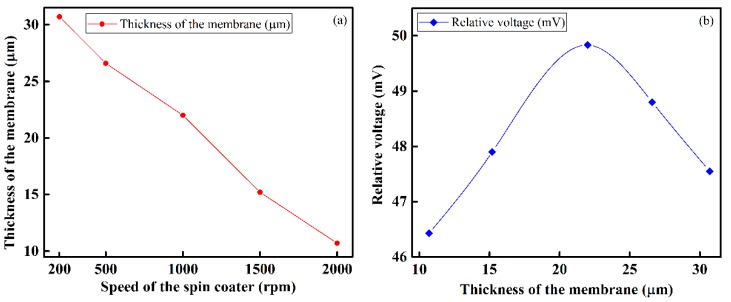
Characteristics of the sensing membrane: (**a**) relationship between the spin coater speed and the thickness of the IDC dielectric membrane; and (**b**) relationship between the thickness of the IDC dielectric membrane of the IDC and the obtained relative voltage.

**Figure 8:**
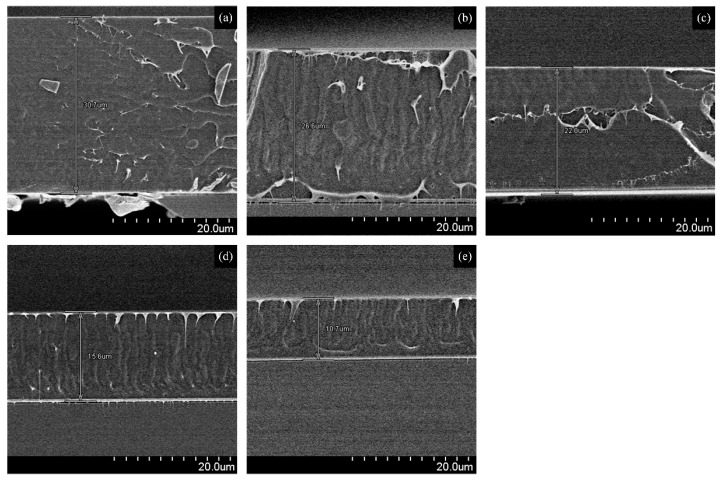
SEM images of different IDC dielectric/sensing membrane thicknesses: (**a**) 30.7 µm; (**b**) 26.6 µm; (**c**) 22.0 µm; (**d**) 15.6 µm; and (**e**) 10.7 µm.

Figure 7a shows the relationship between the speed of the spin coater and the thickness of the dielectric/sensing IDC membrane. As shown, the thickness of sensing membrane is inversely proportional to the speed of the spin coater, and decreases linearly when the speed increases. To optimize the dielectric/sensing membrane thickness, *i.e.*, to find out the specific thickness of the dielectric membrane at which the IDC sensing element offers a maximum relative voltage, we used different thicknesses for this membrane, and measured the relative voltages obtained. The experimental results show that a 22-µm thickness produces the highest relative voltage. Therefore, in our experiment we used sensing membranes with approximately 22 µm, corresponding to a spin coater speed of 1000 rpm. The effect on the relative voltage of a change in the sensing membrane thickness is shown in Figure 7b. Figure 8 shows SEM images of various IDC dielectric/sensing membrane thicknesses.

**Figure 9:**
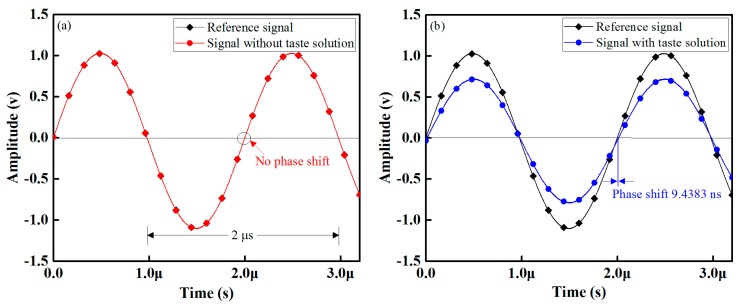
Waveform response of the designed electronic tongue: (**a**) response with reference solution (DI water) and (**b**) response with taste (glucose) solution.

The capacitance, phase shift, and capacitive impedance of the develop IDCs are functions of the taste solution’s concentration. Figure 9a presents the waveforms of the sensing and reference signal when no taste solution is in the test container. As shown, there are no differences in phase shift or in amplitudes in the sensing as well as the reference signals. When we inject a 1 mM glucose (taste) solution into the test container, a phase difference/shift within the sensing signal as well as the reference signal occurs. We used an oscilloscope (TDS3032B, Tektronix, Wilsonville, OR, USA) to measure the phase difference within the signals, and show the resulting waveforms in Figure 9b; the phase difference within the reference and a sensing signal at 1 mM glucose concentration is 9.4383 ns. These waveforms also confirm that the signal processing unit of the designed electronic tongue has sufficient performance to detect little differences in both the amplitude of a received sensing signal and phase difference/shift.

**Figure 10:**
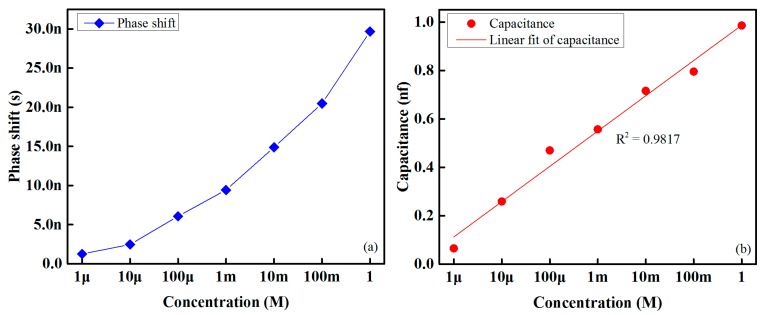
Behavior of the sensing element of the designed electronic tongue for various concentrations of glucose: (**a**) phase shift and (**b**) capacitance.

Figure 10a shows the phase shift within the sensing signal and the reference signal at glucose concentrations ranging from 1 μM to 1 M. As is readily apparent from this figure, the phase difference/shift within the two signals increases linearly with the increase in glucose concentration. The variation of capacitance with respect to the concentration of glucose/taste solution is shown in Figure 10b.

**Figure 11:**
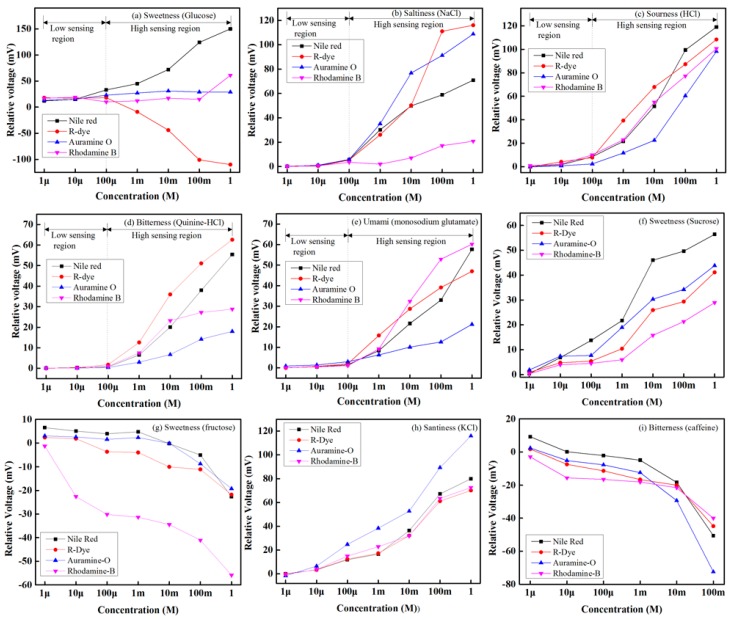
Sensing response of the designed IDC sensing elements for various tastes and concentrations: (**a**) sweetness (glucose); (**b**) saltiness (NaCl); (**c**) sourness (HCl); (**d**) bitterness (quinine-HCl), (**e**) umami (monosodium glutamate); (**f**) sweetness (sucrose), (**g**) sweetness (fructose); (**h**) saltiness (KCl); and (**i**) bitterness (caffeine).

We individually injected various taste solutions—sweetness (glucose), saltiness (NaCl), sourness (HCl), bitterness (quinine-HCl), and umami (monosodium glutamate)—with different concentrations of 1 μM to 1 M into the test container, at room temperature, to see the sensing response of every sensing element of the electronic tongue. The obtained results for all four sensing elements of the array (electronic tongue) are shown in Figure 11 for five different taste solutions. 

The relative voltage of each sensing element is considered to be the response of that specific sensing element of the array. As shown in this figure, the relative voltage rises as the taste solution’s concentration rises. We divided the sensing performance of the array into two regions: the low and high sensing regions. The low sensing region covers concentrations from 1 µM to 100 µM, while the high sensing region covers concentration values of 100 µM to 1 M. As can be seen in Figure 11, every sensing element of the array provided a linear response over its dynamic range. In our study, we obtained that the highest detection performance was consistently achieved with sweetness (glucose solution), while the lowest detection performance was consistently obtained for bitterness (quinine-HCl). The relative voltages of glucose and bitterness at a concentration of 1 M were approximately 150 mV and 18 mV, respectively. We also observe the sensing ability of the proposed electronic tongue under other basic taste solutions, namely: sucrose and fructose for sweet sensation, KCl for salty sensation, and caffeine for bitterness sensation and the results are shown in Figure 11f–i, respectively.

**Figure 12:**
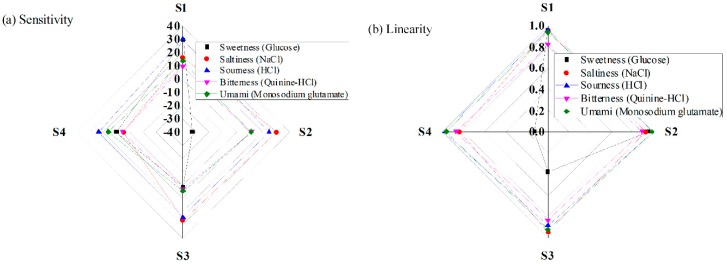
Performance of the designed IDC taste sensor array for various taste solutions: (**a**) sensitivity; and (**b**) linearity.

The radar chart depicted in Figure 12a shows the sensitivity performances of the four IDC taste sensing elements (S1 to S4) under various taste solutions: sweetness, saltiness, sourness, bitterness, and umami. As shown, the first IDC sensing element S1 (which contains the Nile red sensing/dielectric membrane) presents higher sensitivity for sweetness (glucose) and sourness (HCl); the second sensing element S2 (which contains the R-dye dielectric membrane) offers higher sensitivities for saltiness (NaCl) and bitterness (quinine-HCl); the Rhodamine B sensing element S4 shows high sensitivity for umami (monosodium glutamate) only, and the sensing element S3 (Auramine O sensing membrane) shows low sensitivities for most of the taste solutions: sweetness, saltiness, bitterness, and umami. The highest sensitivities of the designed electronic tongue for sweetness, saltiness, sourness, bitterness, and umami are approximately 29.4, 30.64, 29.88, 11.56, and 16.19 mV/decade, respectively.

We considered the sensitivity and linearity performance of the proposed IDC sensor array at the high sensing region (100 µM–1 M) because the human taste threshold levels are mostly within this range; the threshold levels for sweetness, saltiness, sourness, bitterness, and umami are 0.01 M, 0.01 M, 0.0009 M, 0.000008 M, and 0.0007 M, respectively [86]. From the above-presented results, we can therefore tell that the developed electronic tongue can cover the threshold levels of human taste.

The linearity of the designed IDC sensor array/ electronic tongue system under various kinds of tastes is shown in Figure 12b; on the dynamic range of the high sensing region (100 µM–1 M), the designed sensing system offers the highest degree of linearity in the response to the HCl solution, with an *R*^2^ value of approximately 0.985 for the R-dye sensing membrane, and the least linear response appears in the Rhodamine B sensing element when measuring the glucose solution.

To determine the reproducibility response of the designed IDC sensing element of the electronic tongue, we fabricated three samples of IDC sensing elements which contained R-dye sensing membrane. Then we measured the sensing performance of those IDC sensing elements under 1 mM of HCl solution and observe that all three show almost the same sensing response. Therefore, we say that the proposed IDC sensing elements have better reproducibility and the relative standard deviation (RSD) was approximately 0.029.

**Figure 13:**
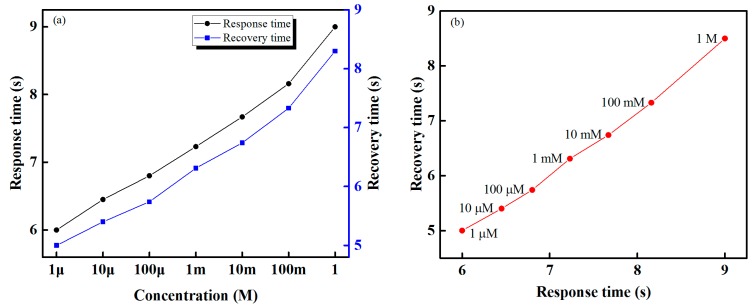
Sensing performance of the designed IDC electronic tongue for different concentrations of HCl: (**a**) response and recovery times and (**b**) response times *versus* recovery times.

The response and recovery times of the developed IDC electronic tongue is shown in Figure 13. The designed sensing system offers rapid response and recovery times of approximately 6 s and 5 s, respectively; as we can see from Figure 13a, the response and recovery times increase as the taste solution’s concentration increases. Figure 13b shows a plot of response times *versus* recovery times for the designed IDC electronic tongue/taste sensor array for HCl concentrations in the range 1 μM to 1 M; as shown, response times are proportional to recovery times.

A simple and efficient multivariate data analysis method (PCA) has been applied for pattern recognition in our experiments. PCA derives a new set of coordinates, ordered by the data variance along those coordinates. It therefore allows a reduction in the number of dimensions of experimental data. Without major information loss, we can consider only the first few new principal coordinates, because they will contain the majority of the data variance. In this way, multi-dimensional data can be mapped onto only two or three axes.

**Figure 14:**
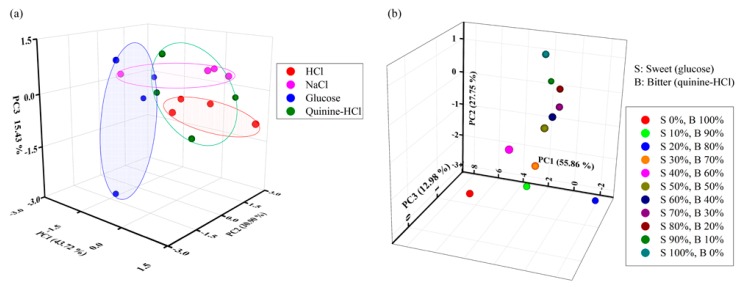
PCA results for the dataset obtained by sensing mixed taste solutions with the designed IDC electronic tongue: (**a**) mixture of four different taste solutions (HCl, NaCl, glucose, and quinine-HCl) and (**b**) different percentages of mixed glucose and quinine-HCl solutions.

In our experiment, we collected the highest relative amplitudes of every array sensing element for five cycles, and constructed an m × n data matrix for PCA, where m = 15 is the number of observations and *n* = 4 is the number of sensing elements. The score plot of the three-dimensional PCA derived principal axis (PC1–PC2–PC3) for sensing discrimination of four different tastes by the developed IDC-electronic tongue is shown in Figure 14a. As shown, the designed IDC taste sensor array/electronic tongue can successfully distinguish various types of tastes from the sensing performance of four sensing elements of the array. To better observe the distinguishing power of the sensor array, we classified the four distinct tastes in the same ellipse and found that PC1 can interpret 43.72% of the variance, whereas PC2 and PC3 can interpret 30.90% and 15.43% of the variance, respectively. Therefore, the overall cumulative variance coverage given by PC1, PC2 and PC3 is 90.05%. From the results shown in Figure 14a, we conclude that the designed IDC-taste sensor array is efficient in successfully distinguishing various kinds of tastes from mixed taste solutions.

**Figure 15:**
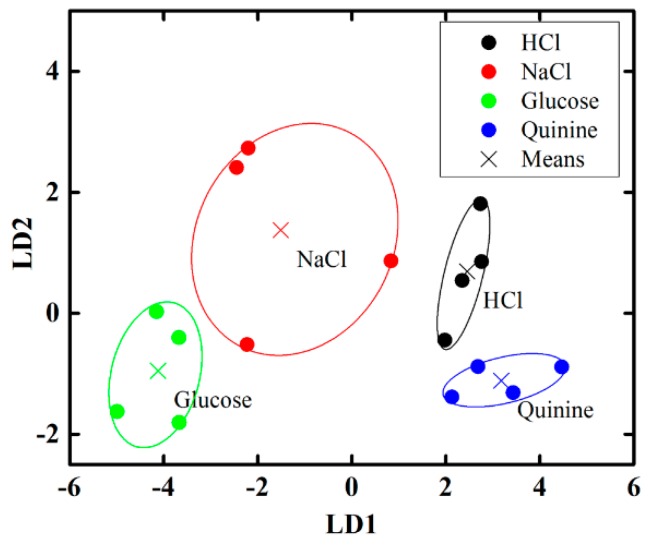
LDA results for the dataset obtained by sensing mixed taste (HCl, NaCl, glucose, and quinine-HCl) solutions with the proposed IDC electronic tongue.

To determine the discrimination performance of the proposed electronic tongue under the mixture of sweet (glucose) and bitter (quinine) compound with different percentages, we applied PCA method and the result is shown in Figure 14b, where it is observed that the electronic tongue can successfully distinguish mixed glucose and quinine solutions of different percentages. In our study, we also have applied the linear discriminant analysis (LDA) method to observe the discrimination performance of the proposed electronic tongue under the mixture of several taste compound such as: HCl, NaCl, glucose, and quinine and the result is shown in Figure 15. According to our analysis we can say that the discrimination performance of the proposed IDC electronic tongue is good and it can differentiate different types of taste from mixtures of different basic taste solutions.

Electronic tongues as well as bioelectronic tongues are hot research topics at the present time [87,88,89]. In [88,89], the authors proposed a bioelectronic tongue to observe taste sensing performance. In their work they fed the taste compound to a rat and collected the taste information from the rat’s gustatory cortex. To do this they inserted a microelectrode array in the rat’s gustatory cortex and coupled the array with the neural network and the array was connected with a signal processor as well as a computer to display taste information. They observed the taste information by collecting signal from the nerves. Though their research is interesting, the detection process is complex and expensive. In our present work, we propose an IDC-based electronic tongue which can directly detect any basic taste as well as complex mixtures of taste substances and display the taste information on a computer monitor. In addition, our sensing system does not need to interface with neurons, which make our sensing system simple in construction and less expensive. We previously proposed a lipid-based IDC taste sensing system to detect different types of tastes [90]. The sensing performance of the lipid-based IDC taste sensing system was good. It featured a multichannel design where each channel has a different lipid containing sensing membrane. Therefore, the lipid-based IDC taste sensing system could simultaneously collect taste information from all sensing elements of the array for a given concentration of the taste solution. The response as well as recovery time were approximately 12.92 s and 13.3 s, respectively. In [91], we reported for the first time an IDC-based glucose biosensing system which had a sensing/dielectric membrane containing a solvatochromic dye. The glucose biosensing system had a single channel, and the proposed IDC glucose biosensing system has a faster sensing response than the lipid-based IDC taste sensing system and its response and recovery times were about 7 s and 5 s respectively. In our present work, we proposed an IDC electronic tongue/taste sensing system, where we used taste sensitive sensing membranes incorporating different solvatochromic dyes to make the IDC array sensing elements. This electronic tongue also has multichannel sensing ability, therefore the proposed electronic tongue could collect taste information at the same time from the all sensing elements. Moreover, the proposed electronic tongue offers a highly sensing response and its response time was about 6 s, which was much shorter than that of the proposed lipid-based IDC taste sensing system as well as shorter than that of the glucose biosensing system.

We also compared the response of the developed solvatochromic sensing membrane IDC electronic tongue/taste sensor with other different sensors, such as potentiometric [92], complementary metal-oxide-semiconductor compatible lateral bipolar transistors (C-CLBT) [27], commercial electronic tongues (SA402) [93,94], and optical fiber sensors [29], in what respects dynamic range width, sensitivity, linearity, reproducibility, and response time. The various sensors’ performances are tabulated in Table 4. As shown in this table, the overall performance of the proposed IDC electronic tongue/taste sensor array compares favorably to the mentioned taste sensors.

## 5. Conclusions

In this paper, for the first time, an interdigitated capacitor (IDC) taste sensor array containing solvatochromic dyes to detect five different kinds of tastes—sweetness, saltiness, sourness, bitterness, and umami—is proposed. Four types of solvatochromic dyes: Nile red, Auramine O, Reichardt’s dye (R-dye), and Rhodamine B, were separately mixed with the polyvinylchloride (PVC) and N,N-dimethylacetamide (DMAC) as solvent, to create four different types of dielectric/sensing solutions, which were then used in the IDC sensing elements. The taste detection system was based on a capacitance variation principle: the IDC capacitance changes due to changes in the concentration of taste solutions and, consequently, the received sensing signal’s amplitude changes as well. The proposed IDC electronic tongue has very short response and recovery times of approximately 6 and 5 s, respectively, and has a wide dynamic range. The sensing performance of the developed electronic tongue were found to be linear, with a 0.985 correlation coefficient (*R*^2^) and the system has high sensing abilities. The designed electronic tongue has many advantages, including low cost, simple construction, the capability to provide real time responses, highly stable sensing performance, and high reproducibility; the components for the required electronic circuitry are inexpensive, and are readily available from any local electronic component market. To observe the discrimination ability of the proposed electronic tongue, a statistical technique (principal component analysis) was applied, and it was found that the designed IDC electronic tongue could successfully classify various kinds of taste from data collected with the sensor array. In future studies, various sensing substances will be applied to increase the number of sensing elements in the array, and we also plan to design IDC-based electronic noses.

## Figures and Tables

**Table 1:** Chemical compounds for taste appreciation categorization.

Taste	Chemical Compounds
Sweet	Sugar, fructose, saccharin, sucrose, glucose, few amino acids, l-alanine, alcohols.
Salt	Metal ions (inorganic salts) : NaCl, KCl, KNO_3_
Sour	Acids (detachment of H+ in solution): HCl, H_2_SO_4_, CH_3_COOH, *etc.*
Bitter	Alkaloids (nicotine, quinine, caffeine) and non-alkaloids (aspirin), MgCl_2_, urea, l-tryptophan, *etc.*
Umami	Amino acids (glutamate)

**Table 2:** Three levels of analogy between human and electronic tongue taste recognition mechanisms.

No. of Stage	Processing Level	Name of the Sensing Mechanism	
		Humans Tongue	Electronic Tongue
1	Receptor level	Buds	Membranes of the sensing elements
2	Circuit level	Neural transmission	Transducer
3	Perceptual level	Cognition in the thalamus	Computer as well as statistical analysis

**Table 3:** Composition of the sensing/dielectric solution for the different sensing elements.

Sensor ID	Solvatochromic Dye	Polymer	Solvent
S1	Nile-red	PVC	DMAC
S2	Reichardt’s dye (R-dye)	PVC	DMAC
S3	Auramine O	PVC	DMAC
S4	Rhodamine B	PVC	DMAC

**Table 4:** Summary of sensing performance for the different sensing systems.

No.	Sensing System	Parameters					Ref.
		Dynamic Range Width	Sensitivity (Umami)	Linearity	Response Time	Reproducibility (RSD)	
1	Proposed Electronic Tongue	1 µM–1 M	16.19 mV/decade	0.985	6 s	0.029	This work
2	Potentiometry	0.1 µM–100 mM	-	poor	> 6.45 min	-	[92]
3	C-CLBT	1 fM–1 mM	12.2 µA/decade	good	-	-	[27]
4	Electronic Tongue (SA402)	1 µM–1 M	13.0 mV/decade	-	20 s	-	[93,94]
5	Optical Fiber	0.1 µM–10 mM	-	-	10 s	poor	[29]
